# Editorial: 15 years of frontiers in human neuroscience: neuromodulation

**DOI:** 10.3389/fnhum.2024.1380491

**Published:** 2024-02-12

**Authors:** Shozo Tobimatsu, Chella Kamarajan

**Affiliations:** ^1^Faculty of Medicine, Neurological Institute, Kyushu University, Fukuoka, Japan; ^2^Department of Psychiatry, Downstate Health Sciences University, Brooklyn, NY, United States

**Keywords:** neuromodulation, brain stimulation, transcrancial magnetic stimulation (TMS), translingual neurostimulation (TLNS), transcranial direct current stimulation (tDCS), transcranial static magnetic stimulation (tSMS), theta burst stimulation (TBS), temporal interference stimulation

Neuromodulation represents purposeful alteration of neuronal properties in order to produce a dynamic and targeted regulation of neuronal circuits and ensuing brain functions (Krames et al., 2018). Neuromodulation plays an important role in the treatment of a variety of neuropsychiatric disorders (Johnson et al., 2013). Over the past few decades, scientific understanding and clinical applications of *neuromodulation* have been on a fast-track (Trapp and Williams, 2021). This editorial attempts to summarize the contributions of the articles published under the Research Topic *neuromodulation*, which is a part of a series that showcases some of the most notable advances in the field of Human Neuroscience over the past 15 years by compiling recent scientific advancements in the field. The topic received three original research articles, one brief research report, and one mini review, which are briefly summarized below.

Kirby et al. investigated translingual neurostimulation (TLNS), a non-invasive and portable neurostimulation device designed for the movement control rehabilitation which is often paired with physical therapy (Diep et al., 2021). The study measured the effects of TLNS on the changes in neurophysiological cognitive measures of event-related potentials (ERP) in patients with mild-to-moderate traumatic brain injury, stroke, Multiple Sclerosis, Parkinson's disease, and other neurological conditions. Interestingly, there were significant variations in ERP components (i.e., N100 and N200) between baseline and endpoint following the TLNS treatment, showing condition-specific significant improvements in attention processing. This study demonstrated objective measures of neuromodulation induced improvement and thus validated TLNS as a useful tool in cognitive rehabilitation.

Shirota et al. examined technical feasibility of using MEG recordings during transcranial Direct Current Stimulation (tDCS) since concurrent use of both technologies remained a challenge due to the fact that tDCS induces artifacts on the MEG signal. The researchers used a phantom device to produce an artificial current dipole simulating focal brain activity. Further, equivalent current dipoles (ECD), a measure of current dipole sources, was estimated and its quality was assessed using localization error, amplitude error, and goodness of fit. ECD modeling performance, with and without tDCS, was also measured. The objective was to see whether implementation of artifact rejection using temporally extended signal space separation (tSSS) could recover the MEG signals recorded during simultaneous application of MEG and tDCS. The findings confirmed that concurrent tDCS-MEG recording is feasible, especially when artifact rejection was performed using the tSSS method. Thus, the study demonstrated that while tDCS does affect MEG signals, artifact rejection techniques such as tSSS can be highly useful to considerably restore the signal. This finding may have huge implications for simultaneous tDCS-MEG research and applications.

In a validation study, Matsumoto et al. investigated the effect of the conventional single Transcranial static magnetic stimulation (tSMS) as well as triple tSMS over the unilateral or bilateral motor association cortex (MAC) on simple and choice reaction time (SRT and CRT) task performance. The main aim of the study was to confirm whether the recently developed triple tSMS device, which had three magnets placed close to each other, can produce stronger and broader static magnetic field than the conventional single tSMS. The study consisted of two experiments: one involved the conventional tSMS, and the other involved the triple tSMS. In both experiments, the participants received tSMS over the unilateral (left) MAC, tSMS over the bilateral MAC, and sham stimulation at the premotor cortex while they performed simple and choice reaction time (SRT and CRT) tasks before, immediately after, and 15 min after the stimulation. The findings revealed that single tSMS over the unilateral or bilateral MAC did not affect performance of RT tasks, whereas triple tSMS over the bilateral MAC but not over the unilateral MAC increased variability of CRT, suggesting that RT task performance could be modulated using triple tSMS.

In a study measuring the effects of type and frequency of theta burst stimulation (TBS) on cognitive processing, Harrington et al. compared these effects in continuous TBS (cTBS) vs. intermittent TBS (iTBS) during task performance. Fourteen young adult participants received either iTBS or cTBS to the supramarginal gyrus while performing a pseudoword discrimination task and an orthographic awareness task at four different time points. While there was no effect of stimulation type in reaction time suggesting that both types of TBS caused similar effects, reaction time was found decreased over time in the pseudoword task, indicating faster pseudoword processing speed with better performance 60–70 min after stimulation. In contrast, no change was observed over time for the behavioral control task, indicating that the change over time seen in the test condition was not a learning effect. These findings provide valuable insight into the effects of stimulation type and frequency (timing) on the linguistic processing in the brain.

Finally, Zhu and Yin performed a comprehensive literature review on the effect of Temporal Interference (TI) stimulation on human performance by reviewing various studies involving preclinical, human, and computer simulations in order to elucidate the mechanism and safety of the TI stimulation paradigm. While TI could stimulate deep motor cortex and induce movement without affecting the overlying cortex, studies are limited to understand and ascertain its efficacy in human trials. The authors have reviewed available studies and identified the gaps in the literature, while suggesting potential future applications and directions. This review affirms that TI is a promising brain stimulation technology for the treatment of neurological movement disorders, due to its superior focality, steerability, and tolerability compared to traditional electrical stimulation. The authors further recommend that since human experiments have yielded fewer and inconsistent results, simulation experiments in animals are still required to optimize stimulation protocols that are to be applied in potential human trials. Thus, the review offers valuable insights into the current status and future requirements for the stimulation protocols pertaining to a viable TI stimulation paradigm.

In sum, each of the five papers offer valuable contributions to the methods, protocols, and potential applications of existing and innovative brain stimulation methods. However, as these papers recommend, more empirical research and human trials are warranted to further optimize stimulation protocols in order to achieve desired therapeutic and clinical outcomes and to expand potential clinical applications of various neuromodulation techniques.

## Author contributions

ST: Writing—review & editing. CK: Writing—original draft, Writing—review & editing.
